# Collagen XII is Commonly Downregulated in the Dermal Extracellular Matrix in Diverse Skin Pathologies

**DOI:** 10.1002/mco2.70282

**Published:** 2025-10-20

**Authors:** Luís Martins, Mariana D. Malta, Sara Chaves, Hugo Osório, Christina Guttmann‐Gruber, Thomas Kocher, Alexandra P. Marques

**Affiliations:** ^1^ 3B's Research Group I3Bs‐Research Institute on Biomaterials Biodegradables and Biomimetics University of Minho Headquarters of the European Institute of Excellence on Tissue Engineering and Regenerative Medicine Guimaraes Portugal; ^2^ i3S – Institute for Research and Innovation in Health IPATIMUP – Institute of Molecular Pathology and Immunology of the University of Porto University of Porto Porto Portugal; ^3^ EB House Austria Research Program for Molecular Therapy of Genodermatoses Department of Dermatology and Allergology University Hospital of the Paracelsus Medical University Salzburg Austria

1

Dear Editor,

Extracellular matrix (ECM) is the pilar of skin structural stability. Several alterations in ECM proteins have been associated with skin disorders of various etiologies, from heritable and autoimmune to skin cancers [1, 2].

In skin blistering diseases such as dystrophic epidermolysis bullosa (DEB), caused by mutations in the *COL7A1* gene [3], and pemphigus vulgaris (PV), induced by auto‐antibodies against DSG1/3 in the epidermis [4], excessive dermal inflammation is a common feature. In both diseases, blistering is associated not only with chronic skin fragility but also with altered ECM organization in DEB [2, 3] and increased matrix metalloproteinases in PV [4], which are directly involved in collagen degradation. Similarly, in cutaneous squamous cell carcinoma (SCC), a skin tumor with abnormal keratinocyte proliferation and dermal invasion [3], up‐regulated enzymes degrade basement membrane components like laminin and collagen IV, correlating with tumor invasiveness. Additionally, the most severe form of DEB is associated with the development of SCC [2, 3].

Recent studies on immune‐mediated skin diseases like vitiligo and psoriasis highlight the critical role of dermal fibroblasts in disease pathogenesis. In vitiligo, fibroblasts define melanocyte‐affected patterns in the epidermis [5], while in psoriasis, their signaling drives basal epidermal cell proliferation [6]. Transcriptomic analysis also revealed shared molecular signatures across various inflammatory skin diseases. Based on this evidence, we hypothesized that identifying common pathogenic mechanisms and therapeutic targets could increase treatment prospects for rare diseases. To explore this, we analyzed the proteome profiles of fibroblasts derived from dominant DEB (DDEB), intermediate recessive DEB (intRDEB), severe recessive DEB (sevRDEB), PV of cutaneous (PVcut), and mucosal (PVmuc) origins, and cutaneous SCC diseased skin, as models for epidermal diseases with distinct pathological origins.

Following liquid chromatography‐mass spectrometry analysis of fibroblasts cultured in over‐confluence to promote ECM deposition, we identified a total of 5220 proteins. Differential expression analysis comparing each disease to the healthy control revealed 1149, 1300, 1333, 107, 276, and 1278 differentially expressed proteins (DEP) in DDEB, intRDEB, sevRDEB, PVcut, PVmuc, and SCC comparisons, respectively (Additional Data).

Enrichment analysis of DEPs and examination of the top 10 GOBP terms associated with each comparison revealed no common terms between them (Additional Data). To get further insight we did enrichment analysis for down‐ and up‐regulated DEPs separately. We found that in the down‐regulated proteins, top terms were associated with ECM and structure organization, and were shared by all DEB and PV variants (Figure 1A). Top GOBP terms associated with up‐regulated DEPs were shared by all DEB variants and SCC and were related to mRNA splicing and processing. Dysregulation in splicing is an important factor in the severity of DEB and in the development of various types of SCC [7]. Notably, DEB patients, especially those suffering from sevRDEB, are more susceptible to developing SCC [3]. Therefore, our results suggest a potential role for RNA splicing dysregulation in the progression of SCC in DEB patients.

**FIGURE 1:**
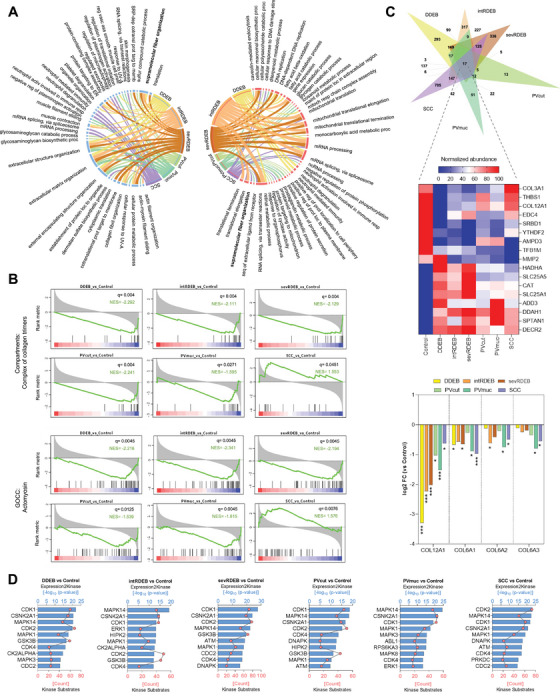
Proteomic profile of dermal fibroblasts across skin diseases. (A) Circos plots showing the overlap of the top 10 Gene Ontology Biological Processes (GOBP) of down‐regulated (left) and up‐regulated (right) differentially expressed proteins (DEP) of each comparison disease vs control. Ribbon size represents ‐log_10_ (*p*‐value)—wider ribbons correspond to higher enrichment. Enrichment analysis was performed using the Enrichr tool separately for down‐ (left) or up‐regulated (right) proteins; (B) Gene Set Enrichment Analysis (GSEA) enrichment plots of the altered Gene Ontology (GO) child terms under GO supramolecular fiber organization term for each comparison disease vs control. Black vertical lines represent ranked proteins in the listed signatures. The green curve represents the Normalized Enrichment Score (NES). The more the green line is shifted to the upper left side of the graph the more the signature is enriched in the disease. In contrast, when the green line shifts to the lower right side the higher the enrichment on the control. The red‐to‐blue colored scale at the bottom represents the degree of correlation of protein's abundance in the disease group (red: positive; blue: negative correlation) or vice versa for the control; (C) Venn diagram showing the intersection of significantly down‐ and up‐regulated proteins (cut‐off value: fold change ± 0 and q‐value < 0.05) of all comparisons of diseases versus control (top); Normalized heatmap of the 17 proteins commonly altered in all disease variants. For each protein dark red (100) and dark blue (0) corresponds to the highest and lowest abundance, respectively, for that specific protein (middle); log2 Fold Change expression (disease vs control) of COL12A1, COL6A1, COL6A2, and COL6A3. Asterisks denote (LIMMA q‐value) significance against control (*q < 0.05, **q < 0.01, and ***q < 0.001) (bottom); (D) Bar plots showing the putative top 10 most significant kinases (sorted by significance level, *p*‐value < 0.05) upstream of DEPs of each disease variant. Kinases were deduced using the Kinase enrichment analysis module of the eXpression2Kinase web tool.

Interestingly, the enrichment analysis also showed that supramolecular fiber organization GOBP was associated with SCC up‐regulated DEPs and with DDEB, intRDEB, sevRDEB, and PVcut down‐regulated DEPs. To confirm the association of this GO term with all diseases, we carried out a pre‐ranked Gene Set Enrichment Analysis testing gene set signatures related to supramolecular fiber organization child terms. The only significantly enriched signatures in all comparisons were complex collagen trimers and actomyosin (Figure 1B). These two gene sets showed negative normalized enrichment scores (NES) for all DEB and PV variants, while NES was positive for SCC, showing that collagen and myosin‐related processes are altered in all diseases but not in the same manner.

To investigate which common proteins are potentially involved in disease alterations, we performed an intersection analysis of all DEPs of each comparison (Figure 1C, top). Only 17 proteins were significantly altered in all the diseases (Figure 1C, middle). COL3A1, one of the main collagens in the skin ECM, was the only common protein down‐regulated in DEB and PV while up‐regulated in SCC, an expression pattern shared by other collagens, myosins, and tropomyosins, although not consistent with statistical significance (Additional Data). Notably, COL12A1 was significantly down‐regulated in all diseases, while COL6A1, COL6A2, and COL6A3 were down‐regulated, although not significantly (Figure 1C, bottom).

This is noteworthy since defects in collagen XII and VI have been linked to ECM alterations in various tissues, including the skin in myopathic Ehlers‐Danlos syndrome, a condition characterized by skin hyperextensibility and tissue fragility. Extracellularly, collagen XII interacts with collagen I fibrils, regulating fibril spacing and assembly. It also interacts with other ECM molecules involved in fibrillogenesis, tissue structure, and mechanotransduction, responding to mechanical stress cycles [8]. Moreover, collagen XII mediates the progression and metastatic potential in gastric cancer and esophageal SCC, the latter being also characterized by dispersed collagen fibers [9]. Recent studies on RDEB patient samples have revealed alterations in ECM organization, and reduced collagen XII secretion, despite its increased intracellular expression and accumulation. As for PV, to the best of our knowledge, no direct targets in the dermal layer have been described. However, considering our findings and evidence from other immune‐mediated skin diseases, where dermal fibroblasts were identified as responsible for the pattern of immune activity in the epidermis [5, 6], it is worth exploring if regions with impaired collagen fibril assembly and organization, possibly caused by collagen XII dysregulation, may be more vulnerable to PV autoantibodies targeting. As such, we speculate that collagen XII dysregulation may play a common role in mediating the pathogenesis of the studied skin diseases. Nevertheless, future investigation is necessary to elucidate the importance of collagen XII compartmental alterations in ECM organization, collagen trimer dynamics, and actomyosin processes.

Next, we aimed to identify a possible common upstream regulatory environment responsible for the observed protein expression. We used the eXpression2Kinases network algorithm to perform a kinase enrichment analysis feeding DEPs and predicted transcription factors as inputs. It consistently identified cyclin‐dependent kinases (CDK1 and CDK4), mitogen‐activated protein kinases (MAPK1 and MAPK14), and casein kinase (CSNK2A1) among the top‐enriched kinases for all diseases (Figure 1D), suggesting that the alterations may stem from the activity of shared upstream regulators. Several studies highlight MAPK pathways mediation in the deposition of ECM in various cell types, including dermal fibroblasts [10]. Strikingly, recent evidence demonstrated that MAPK signaling mediates cell migration, proliferation, and metastasis through collagen XII in several cancers [9].

Importantly, the exploratory nature of our study, with a small number of samples, needs to be considered. Additionally, while our over‐confluence cell model enables the study of ECM alterations, the possible impact of cell density and mechanical strain on cell response and ECM composition should also be contemplated.

Ultimately, despite the disparities in the dermal fibroblasts’ proteome of skin diseases from different etiologies, our study highlights a common pattern of collagen XII dysregulation that may contribute to impairment of ECM complex of collagen trimer and actomyosin processes, potentially leading to skin fragility, blistering, and carcinogenic phenotypes. We believe our findings provide a foundation for future, more comprehensive investigations on the relationship between dermal fibroblasts collagen XII expression and secretion, and its regulatory mechanisms in the pathology of skin diseases.

## Author Contributions

L.M., M.D.M., and S.C. conducted cell experiments. H.O. conducted LC‐MS/MS experiments. C.G‐G. and T.K. provided the DEB cells. L.M. and A.P.M. designed the study, analyzed the data, and wrote the manuscript. A.P.M. provided funding.

## Ethics Statement

In this study, sample collection procedures were performed after informed consent at Hospital São João (Porto, Portugal), complying with ethical regulations regarding research involving human participants as approved by the Ethical Committee of Hospital São João (169/17; 477/2020) and Ethical Committee for Research in Life and Health Sciences of the University of Minho (CEICVS 135/2020). DEB E6/E7 HPV‐immortalized (415‐EP/73/192‐2013 and 415‐E/2118/9‐2017) hdFbs were provided by EB House Austria (MTA/2017/05/23).

## Conflicts of Interest

The authors declare no conflicts of interest.

## Supporting information




**Supporting File 1**: mco270282‐sup‐0001‐SuppMat.pdf

## Data Availability

Raw data can be available upon reasonable request upon publication. Additional Data is available at: https://doi.org10.5281/zenodo.15412418

## References

[1] M. D. Malta , M. G. Fernandes , L. Martins , et al. Implications of Self‐assembled Extracellular Matrix for Dermal‐epidermal Interactions in Health and Disease. NANOTODAY‐D‐24‐00949, Available at SSRN. 10.2139/ssrn.4869019

[2] M. D. Malta , M. T. Cerqueira , A. P. Marques , “Extracellular Matrix in Skin Diseases: The Road to New Therapies,” Journal of Advanced Research 51 (2023): 1‐14. 36481476 10.1016/j.jare.2022.11.008 PMC10491993

[3] V. R. Mittapalli , J. Madl , S. Löffek , et al., “Injury‐driven Stiffening of the Dermis Expedites Skin Carcinoma Progression,” Cancer Research 76, no. 4 (2016): 940‐951. 26676755 10.1158/0008-5472.CAN-15-1348

[4] N. Cirillo , M. Lanza , L. Rossiello , F. Gombos , A. Lanza , “Defining the Involvement of Proteinases in pemphigus vulgaris: Evidence of Matrix Metalloproteinase‐9 Overexpression in Experimental Models of Disease,” Journal of Cellular Physiology 212, no. 1 (2007): 36‐41. 17311292 10.1002/jcp.20997

[5] Z. Xu , D. Chen , Y. Hu , et al., “Anatomically Distinct Fibroblast Subsets Determine Skin Autoimmune Patterns,” Nature 601, no. 7891 (2022): 118‐124. 34912121 10.1038/s41586-021-04221-8

[6] J. Jiang , X. Shao , W. Liu , et al., “The Mechano‐chemical Circuit in Fibroblasts and Dendritic Cells Drives Basal Cell Proliferation in Psoriasis,” Cell Reports 43, no. 7 (2024): 114513. 39003736 10.1016/j.celrep.2024.114513

[7] D. F. Murrell , J. Uitto , “Splicing of the Cake Can Affect the Severity of Epidermolysis Bullosa,” Journal of the European Academy of Dermatology and Venereology 34, no. 9 (2020): 1879‐1880. 33448527 10.1111/jdv.16859

[8] Y. Izu , D. E. Birk , “Collagen XII Mediated Cellular and Extracellular Mechanisms in Development, Regeneration, and Disease,” Frontiers in Cell and Developmental Biology 11 (2023): 1129000. 36936682 10.3389/fcell.2023.1129000 PMC10017729

[9] J. Li , Z. Li , Y. Xu , C. Huang , B. Shan , “METTL3 facilitates Tumor Progression by COL12A1/MAPK Signaling Pathway in Esophageal Squamous Cell Carcinoma,” Journal of Cancer 13, no. 6 (2022): 1972‐1984. 35399719 10.7150/jca.66830 PMC8990406

[10] L. Zhu , L. Liu , A. Wang , J. Liu , X. Huang , T. Zan , “Positive Feedback Loops Between Fibroblasts and the Mechanical Environment Contribute to Dermal Fibrosis,” Matrix Biology 121 (2023): 1‐21. 37164179 10.1016/j.matbio.2023.05.001

