# Supplementary material for: Collagen XII is Commonly Downregulated in the Dermal Extracellular Matrix in Diverse Skin Pathologies
Source: MedComm (2020). 2025 Oct 20;6(11):e70282. doi: 10.1002/mco2.70282 (PMC12538003; doi:10.1002/mco2.70282)
Supplement: Supplementary file 1 — Supporting File 1: mco270282‐sup‐0001‐SuppMat.pdf [file MCO2-6-e70282-s001.pdf]

Supporting Information

Collagen XII is Commonly Downregulated in the Dermal Extracellular Matrix in Diverse Skin Pathologies

Authors:

Luís Martins[1,*], Mariana D. Malta[1], Sara Chaves[1], Hugo Osório[2], Christina Guttmann-Gruber[3], Thomas Kocher[3], Alexandra P. Marques[1,*]

Affiliations:

[1] 3B's Research Group, I3Bs-Research Institute on Biomaterials, Biodegradables and Biomimetics, University of Minho; Headquarters of the European Institute of Excellence on Tissue Engineering and Regenerative Medicine; ICVS/3B's-PT Government Associate Laboratory; 4805-694 Guimarães, Portugal

[2] i3S – Institute for Research and Innovation in Health; IPATIMUP – Institute of Molecular Pathology and Immunology of the University of Porto; University of Porto, 4200-135 Porto, Portugal

[3] EB House Austria, Research Program for Molecular Therapy of Genodermatoses, Department of Dermatology and Allergology, University Hospital of the Paracelsus Medical University, 5020 Salzburg, Austria

* Correspondence: luis.martins@i3bs.uminho.pt (L.M.), apmarques@i3bs.uminho.pt (A.P.M.)

MATERIALS AND METHODS

Fibroblast isolation and culture

Healthy skin samples were collected from discarded tissue from adult patients who underwent abdominoplasty surgical procedures. Cutaneous and mucosal PV and cutaneous SCC skin samples were collected from patients undergoing pre-diagnostic biopsy procedures. Cutaneous PV (PVcut) sample was collected from the patient forearm skin blister. Mucosal PV (PVmuc) sample was obtained from the patient oral mucosa blister. SCC samples were collected from epidermal lesions, moderately differentiated, with up to 2mm invasion thickness.

Biological sample collection was performed after informed consent at Hospital São João (Porto, Portugal), complying with ethical regulations regarding research involving human participants as approved by the Ethical Committee of Hospital São João (169/17; 477/2020) and Ethical Committee for Research in Life and Health Sciences of the University of Minho (CEICVS 135/2020). DEB E6/E7 HPV-immortalized (415-EP/73/192-2013 and 415-E/2118/9-2017) hdFbs were provided by EB House Austria (MTA/2017/05/23). The mutation profile of DEB variants is as follows. DDEB: c. 6127G>A / --; Exon E73; premature termination codon. intRDEB: 844115del20 / 85058506dupCG; Exon E115; in-frame deletion. sevRDEB: c.425A>G / c.425A>G; Exon E3; Glycine substitution.

Healthy, PV and SCC primary human dermal fibroblasts (hdFbs) were isolated from skin samples using the Whole Skin Dissociation Kit (Miltenyi Biotec), which was developed for the isolation and expansion of fibroblasts, according to the manufacturer's instructions. During the process of isolation and culture no other cells were selected or excluded. Cells were maintained in High Glucose

Dulbecco's modified Eagle's medium (DMEM, Thermo Fisher Scientific) supplemented with 10% FetalClone III serum (FCIII, Hyclone), 1% L-glutamine (Thermo Fisher Scientific), and 1% antibiotic/antimycotic (Thermo Fisher Scientific), and cultured in a humidified incubator at 37ºC ad 5% CO_2. For the experiments, hdFbs ($50x10^3/cm^2$) in passage P4-P6 were cultured at over-confluence in medium supplemented with 50μg/mL ascorbic acid (FUJIFILM Wako Chemicals) for 14 days to promote maximum ECM deposition.

LC-MS/MS sample preparation

Samples were lysed with lysis buffer – 100mM Tris-HCl pH 7.6, 4% sodium dodecyl sulphate (Sigma Aldrich), 100mM dithiothreitol (Sigma Aldrich, and protease inhibitor cocktail (Abcam). Lysates were homogenized with ultrasounds on ice until the buffer solution was clear (3 cycles of 2 seconds each with intervals of 1 minute). Protein concentration of the lysate was measured using the Pierce Coomassie Protein Assay Kit (Thermo Fisher Scientific). 100μg of protein sample was processed following the solid-phase-enhanced sample preparation (SP3) protocol to remove all the components of the lysis buffer.

LC-MS/MS data acquisition

Protein samples were analysed on an Ultimate 3000 liquid chromatography system coupled to a Q-Exactive Hybrid Quadrupole-Orbitrap mass spectrometer (Thermo Scientific) as described elsewhere. Proteome Discoverer (version 2.5.0.400, Thermo Scientific) was used to process MS raw files. Protein identification analysis was performed with the data available in the UniProt protein sequence database for the Homo sapiens Proteome 2021_03 with 20,371 entries

and a common contaminant database from MaxQuant (version 1.6.2.6, Max Planck Institute of Biochemistry). Two protein search algorithms were considered: 1) the mass spectrum library search software MSPepSearch, with the NIST huma HCD Spectrum Library (1,127,970 spectra) and 2) the Sequest HT tandem mass spectrometry peptide data base search program. An ion mass tolerance of 10 ppm for precursor ions and 0.02 Da for fragment ions was considered in both search nodes. The maximum number of allowed missing cleavage sites was set to 2. Cysteine carbamidomethylation was defined as a constant modification and peptide confidence was set to high. The Inferys rescoring node was considered for the analysis. The processing node Percolator was enabled with the following settings: maximum delta Cn 0.05; Target False Discovery Rate-FDR 1%; validation based on q-value. Protein-label-free quantification was performed with the Minora feature detector node at the processing step. Precursor ion quantification was performed at the processing step using the following settings; Peptides: unique plus razor; precursor abundance based on intensity; normalization mode was based on a t-test (background based). A chromatographic retention time alignment was applied using a maximum shift of 10 min and 10 ppm of mass tolerance allowing for mapping features from different sample files. The minimum signal-to-noise (S/N) threshold for feature linking and mapping, was set to 5.

The MS raw data files were filtered by the Proteome Discoverer software node "Spectrum Selector" with the following criteria: General Settings - Precursor Selection: Use MS1 precursor; Provide Profile Spectra: Automatic. Spectrum Properties Filter - Min. precursor mass: 350 Da; Max. Precursor mass: 5000 Da; Minimum peak count: 1. Scan Event Filters - Mass Analyzer: Any; MS Order: Is

Not MS1; Activation Type: Any; Min. Collision Energy: 0; Max. Collision Energy: 1000; Scan Type: Is Full; Polarity Mode: Any.

The accuracy of protein identification was accessed by the Percolator node. This algorithm uses a semi-supervised method to train a machine learning algorithm called a support vector machine to discriminate between positive and negative PSMs. The Percolator parameters were: Target/Decoy Strategy - Target/Decoy Selection: Concatenated; Validation based on: q-value. Input Data - Maximum Delta Cn: 0.05; Maximum rank: 0. FDR Targets - Target FDR (strict): 0.01; Target FDR (Relaxed): 0.05.

Sample replicates from each biological condition were grouped with their chromatographic peaks and features detected by Label Free Quantification using the Minora node. In addition, chromatographic alignment and feature linking and mapping was performed with the Feature Mapper node followed by Precursor Ions Quantification.

Bioinformatics data analysis and visualization

Raw read counts were imported into *Omics Playground* (version v2.8.19, BigOmics Analytics) implemented in *Docker Desktop* (Docker Inc). *Omics Playground* suite was used to perform the following analysis: Differential expression analysis was performed using the trend.LIMMA method and cut-off values specified in the figure legend. Gene set functional enrichment was performed using the Gene Set Enrichment Analysis *test signature* using *fgsea*.

Biological processes enrichment analysis of differentially expressed proteins was performed using the Gene Ontology Biological Processes database implemented on the *Enrichr* web tool.

Volcano plots were generated using the *VolcaNoseR* web tool.

GraphPad Prism (version 9.3.1) was used to generate normalized heatmaps, individual protein expression plots and kinase enrichment plots.

Circos plots were generated using *Circos Table Viewer* (v0.63, Martin Krzywinski) by overlapping the top 10 enriched GOBPs from up-regulated or down-regulated DEPs of each comparison disease versus control.

Venn diagram was generated using the *jvenn* diagram viewer (INRA).

Transcription factors and kinase enrichment analysis

Upstream kinases and transcription factors that are likely to regulate differentially expressed proteins identified were computationally inferred using the *eXpression2Kinases X2K Web* tool. Top DEPs were uploaded into X2K Web which uses an enrichment algorithm that computes a differential expression signature to predict and rank probable transcription factors responsible for the regulation of the interrogated DEPs. X2K Web then builds a network of protein-protein interactions that is fed into the Kinase Enrichment Analysis feature.

Experimental design and statistical analysis

The number samples used in this study was 13 healthy control samples (4-5 replicates from 3 donors), 3 DDEB samples (3 replicates from 1 donor), 3 intRDEB samples (3 replicates from 1 donor), 3 sevRDEB samples (3 replicates from 1 donor), 4 PVmuc samples (4 replicates from 1 donor), 4 PVcut samples (4 replicates from 1 donor), 5 SCC samples (1 sample from 5 donors).

Due to the limited availability of DEB patient samples, in this study we used human DDEB, intRDEB and sevRDEB immortalized fibroblasts instead of

primary fibroblasts. Recently, we have shown that these cells are suitable models for the study of DEB ECM and that the immortalization process did not affect ECM-related processes [1].

Statistical tests and significance levels were applied as part of the bioinformatic analysis in Omics Playground as described in the figure legend.
